# (Ligno)Cellulose Nanofibrils and Tannic Acid as Green Fillers for the Production of Poly(vinyl alcohol) Biocomposite Films

**DOI:** 10.3390/polym17010016

**Published:** 2024-12-25

**Authors:** Urša Osolnik, Viljem Vek, Miha Humar, Primož Oven, Ida Poljanšek

**Affiliations:** Department of Wood Science and Technology, Biotechnical Faculty, University of Ljubljana, Jamnikarjeva 101, 1000 Ljubljana, Slovenia; ursa.osolnik@bf.uni-lj.si (U.O.); viljem.vek@bf.uni-lj.si (V.V.); miha.humar@bf.uni-lj.si (M.H.); primoz.oven@bf.uni-lj.si (P.O.)

**Keywords:** (ligno)cellulose nanofibrils, biocomposite films, tannic acid, poly(vinyl alcohol), mechanical properties

## Abstract

This study compared the use of cellulose nanofibrils (CNF) and lignocellulose nanofibrils (LCNF) in different concentrations to reinforce the poly(vinyl alcohol) (PVA) matrix. Both nanofillers significantly improved the elastic modulus and tensile strength of PVA biocomposite films. The optimum concentration of CNF and LCNF was 6% relative to PVA, which improved the tensile strength of the final PVA biocomposite with CNF and LCNF by 53% and 39%, respectively, compared to the neat PVA film. The addition of LCNF resulted in more elastic films than the addition of CNF to the PVA matrix. The elongation at break of the PVA biocomposite with 2% of LCNF was more than 100% higher than that of the neat PVA film. The integration of tannic acid (TA) into the PVA-LCNF system resulted in antioxidant-active and more water-resistant PVA biocomposites. The three-component biocomposite films with 2 and 6% LCNF and 10% TA exhibited a more than 20° higher contact angle of the water droplet on the surfaces of the biocomposite films and absorbed more than 50% less water than the neat PVA film. New formulations of biocomposite films have been developed with the addition of LCNF and TA in a polymeric PVA matrix.

## 1. Introduction

Packaging materials based on synthetic polymers derived from fossil fuels have a negative impact on human health and the environment as they are non-degradable and accumulate in nature [1,2]. The development and production of alternative packaging materials that are both biodegradable and environmentally friendly has recently increased dramatically, with active packaging materials playing a special role [3,4].

One of the most interesting biodegradable polymers that can be used as a packaging material is poly(vinyl alcohol) (PVA). PVA is an environmentally friendly, water-soluble synthetic polymer with excellent film-forming properties and good mechanical and oxygen barrier properties. The disadvantage of this polymer is its hydrophilicity since its mechanical properties deteriorate when exposed to higher humidity. There are two main ways of increasing the hydrophobicity of PVA. First, PVA can be blended with hydrophobic matrices, and second, nanofillers can be integrated into the PVA matrix. For the production of environmentally friendly and biodegradable PVA nanocomposites, the nanofillers must be compatible with the PVA, biodegradable, and non-toxic [2,5]. One of the solutions is thus to produce PVA biocomposite films in which PVA is crosslinked with other composite components via OH groups so that there are fewer free OH groups in the PVA structure. Additionally, this means that the final composite is less water soluble than the original PVA polymer, and even hydrophobic PVA composites can be obtained [6].

Nanocellulose in the form of nanofibrils [7,8,9] and nanocrystals (CNC) [1,10,11] can be used as an alternative nanofiller to petroleum-based materials in packaging, adhesives, and films. Nanocellulose and tannic acid (TA) are components that could be used to produce biodegradable, antioxidant-active, and mechanically reinforced biocomposites. Our study was based on the integration of lignocellulose nanofibrils (LCNFs) and cellulose nanofibrils (CNFs) into a PVA matrix to produce PVA–nanocellulose biocomposite films. Both LCNFs and CNFs have excellent mechanical and optical properties, high stiffness, a large surface area, and are biodegradable. Because of these unique properties, the integration of LCNFs and CNFs into a polymer matrix could lead to high-value-added products/biocomposites.

In recent years, LCNFs have become very interesting because of their easy availability, higher yields, lower environmental impact, and lower cost compared to CNFs [12,13,14,15]. LCNFs can be produced directly from unbleached pulp and raw lignocellulosic biomass without delignification, which means that LCNF production is more environmentally friendly than CNF production. Due to the residual lignin in LCNFs, these fibrils have some advantages over CNFs, such as higher hydrophobicity, UV-blocking properties, and high antioxidant activity, as well as antibacterial activity against *E. coli* [2,15,16]. The thermal stability of LCNFs compared to CNFs depends on the production process of the two nanocelluloses. Some sources claim that the presence of lignin improves thermal stability [13,17,18], while others state that CNFs have better thermal stability than LCNFs [7]. LCNFs can be crosslinked with other polymer structures, producing different types of composites, films [2,13,19,20,21,22] and hydrogels [12,23], resulting in higher composite strength and stiffness.

There are only a few studies in the literature in which PVA composite films were produced with LCNFs as a reinforcing agent. We found no study in which LCNFs were isolated from spruce wood by treatment with maleic anhydride and subsequently incorporated into a PVA matrix. Espinosa et al. [19] used LCNFs to produce PVA-LCNF composites isolated from wheat straw by two different pretreatments: mechanical pretreatment of the pulp in a PFI beater and TEMPO-mediated oxidation with further mechanical processing using a high-pressure homogenizer. Yang et al. [13] also produced LCNFs from wheat straw by using an acidic hydrotrope of p-toluene sulfonic acid in combination with further ultrasonic treatment at different output powers. Horseman et al. [7] produced composites with LCNFs, but in this study, LCNFs were the base film to which additives such as PVA, CNFs, CNCs, and bentonite were used. Zhang et al. [2] used TEMPO-oxidized LCNFs as a nanofiller to reinforce the PVA matrix to prepare PVA nanocomposite films with improved mechanical and thermal properties and improved water resistance. Bascon Villegas [21] also produced PVA-LCNF nanocomposite films, in which LCNFs were obtained by mechanical and chemical pretreatment (TEMPO-mediated oxidation) of residues from vegetable production.

Similar to LCNFs, tannic acid is a green filler, like many others [24], that has antimicrobial activity and can also reinforce the polymer matrix [8]. TA is a polyphenolic compound with various biological activities [25] and could act as a crosslinker in different polymers, such as PVA [8,26] and chitosan [27,28,29].

We have not found any study dealing with the production of three-component PVA biocomposite films by simultaneous incorporation of LCNFs and TA. With the inclusion of LCNFs, we aimed to produce mechanically reinforced PVA biocomposite films and with the addition of TA, we aimed to investigate the antioxidant activity of TA in bound form when incorporated into the PVA-LCNF system. In a previous study, we found that TA is antioxidant active both in free form and also when incorporated into a PVA-CNF system. The present study is a continuation of the research in which we investigated the effect of LCNFs as a mechanical reinforcement, as opposed to CNFs, in the presence of the antioxidant TA. The main task was to investigate how LCNFs affect the incorporation of TA into the PVA-LCNF system and to study the antioxidant activity of TA in bound form.

## 2. Materials and Methods

### 2.1. Materials

Cellulose nanofibrils (CNFs) were provided by the Centre for Biocomposite and Biomaterial Processing, University of Toronto, Canada. The CNFs were in the form of a water suspension with a solid content of 1.40%. The suspension of CNFs was obtained by mechanical homogenization of sulfite softwood pulp [30]. The material for the production of LCNFs was obtained from commercial spruce wood and processed in a workshop of the Department of Wood Science and Technology. The material was then chopped in a Retsch SM 2000 cutting mill (Retsch, Düsseldorf, Germany) with a sieve with an opening of 1 mm. Maleic anhydride, poly(vinyl alcohol) (PVA), Mw~47,000, tannic acid (TA), 2,2-diphenyl-1-picrylhydrazyl (DPPH), and HPLC grade water were provided by Merck (Sigma-Aldrich Chemie, Taufkirchen, Germany).

### 2.2. Production of LCNFs

LCNFs were prepared as described [31] with some modifications. Five grams of wood flour were mixed with 25 g of maleic anhydride in an Erlenmayer flask. The conical flask was sealed with a watch glass and placed in an oil bath at 130° C for 3.5 h. The esterification reaction took place in the maleic anhydride melt. At the end of the reaction, the reactants were washed with acetone and distilled water. The esterified product was subsequently soaked in 0.5 M NaOH for 5 min to neutralize the carboxylic acid and to obtain a COO- on the surface of the prepared nanofibrils, which reduced the energy consumption in the further mechanical process. The product was washed with distilled water until the pH of the filtrate was neutral. Mechanical fibrillation was performed using a PandaPLUS 2000 high-pressure homogenizer (GEA NS, Parma, Italy). The fiber suspension was passed through the homogenizer three times at 800 bar, and an LCNF water suspension with a 0.7% solid content was obtained.

### 2.3. Film Preparation

PVA biocomposite films were prepared by solvent casting, as described in a previous study [8], using a 10% PVA solution, a suspension of CNFs, a suspension of LCNFs, and TA. Suspensions with different weight percentages of CNFs, LCNFs, and TA relative to the PVA were prepared and treated with an ultrasound probe. The resulting suspensions were then poured into polystyrene Petri dishes. The films were dried for one week at ambient conditions. The compositions of the films and film labels are listed in Table 1. A PVA reference film was also prepared and served as a reference.

### 2.4. Characterization

#### 2.4.1. Thickness

The thickness of the PVA reference film and PVA biocomposite films with CNFs, LCNFs, and TA was measured with a digital vernier caliper (BOCHEM Instrumente GmbH, Weilburg, Germany) with an accuracy of ±0.03 mm. The thickness results are given as an average of 10 measurements for each film.

#### 2.4.2. Morphology

The morphology of the LCNFs and PVA biocomposite films with CNFs, LCNFs, and TA was studied by field emission scanning electron microscopy (FE-SEM; Zeiss ULTRA Plus, Zeiss, Oberkohen, Germany) at an accelerating voltage of 2 kV. An LCNF film and freeze-dried LCNFs were obtained for the analysis of LCNFs. The freeze-dried LCNFs and all films were cut and attached to aluminum supports with carbon tape coated with 5 nm and 10 nm thick platinum.

#### 2.4.3. FTIR Analysis

The chemical structure and the interactions among the components of the PVA biocomposite films were investigated using Fourier transform infrared spectroscopy (FTIR) in ATR mode (attenuated total reflectance). The films were scanned with a Spectrum Two UATR FT-IR instrument (Perkin Elmer, Waltham, MA, USA) with a resolution of 4 cm^−1^ in a spectral range between 4000 and 400 cm^−1^, with 16 scans per film. The spectra were ATR and baseline corrected and normalized.

#### 2.4.4. Tensile Test

The tensile properties (elastic modulus—E_t_, tensile strength—σ_M_, and elongation at break—ε_tb_) of the PVA reference film and PVA biocomposite films with CNFs, LCNFs, and TA were determined on a Zwick/Roell Z005 (Zwick GmbH & Co. KG, Ulm, Germany) universal machine at ambient conditions according to a modified test standard ASTM D882—02 Standard Test Methods for Tensile Properties of Thin Plastic Sheeting) [32]. Specimens for the tensile test were prepared and conditioned prior to testing (23 °C, 50% RH), as described in a previous study [8]. At least 10 specimens were tested for each film.

#### 2.4.5. Thermal Analysis

Thermal analysis was performed on the PVA reference film and the PVA biocomposite films with CNFs, LCNFs, and TA, as previously reported. In brief, thermogravimetric analysis (TGA) of the films was performed using a TG Mettler Toledo TGA/DSC1 instrument (Mettler Toledo, Greifensee, Switzerland). Amounts of 5.0 to 7.0 mg of the films were weighed into 150 µL crucibles and then heated in a temperature range of 25 °C to 600 °C at a heating rate of 10 K/min under an Ar flow of 50 mL/min. Differential scanning calorimetry (DSC) was performed using a Mettler Toledo DSC1 instrument (Mettler Toledo, Greifensee, Switzerland) in an Ar atmosphere at a flow rate of 50 mL/min. The films were heated twice in succession from 0 to 230 °C at a heating rate of 10 K/min and cooled in between, in the same temperature range, at a rate of 5 K/min. Approximately 3.5 to 5.0 mg of the films were placed in 40 μL aluminum crucibles with perforated lids. The degree of crystallinity of the PVA was calculated using the following equation:

(1) $$X_{c}^{DSC}\left[\%\right]=\frac{{\Delta H}_{f}}{{\Delta H}_{f}^{{}^{\circ}}}\times 100$$

where ${\Delta H}_{f}$ and ${\Delta H}_{f}^{{}^{\circ}}$ are the enthalpy of melting of the PVA reference film or the PVA composite film and PVA with 100% crystallinity in J/g. The melting enthalpy for PVA with 100% crystallinity (${\Delta H}_{f}^{{}^{\circ}}$) is 138.7 J/g [33]. The baseline was subtracted in all measurements.

#### 2.4.6. Contact Angle

Hydrophilicity and the reduction in hydrophilicity of the PVA reference and PVA biocomposite films with CNFs, LCNFs, and TA were evaluated by measuring the dynamic contact angle between a water droplet (5 µL) and the surface of the PVA-LCNF-TA biocomposite films over 60 s. The tests were performed on a Theta optical goniometer (Biolin Scientific Oy, Espoo, Finland) with OneAttension version software (https://www.bf.uni-lj.si/en/units/wood-science-and-technology/research/research-equipment/90/) (Biolin Scientific) using the sessile drop method. The results are given as an average of 10 measurements for each film type.

#### 2.4.7. Water Uptake

The water uptake test of the PVA reference film and PVA biocomposite films with CNFs, LCNFs, and TA was evaluated as previously described [8]. The films were cut into 15 mm × 15 mm pieces, dried to the absolute dry constant, and then immersed in a beaker of distilled water for 1 h. After 1 h, the films were wiped with a paper towel and weighed again. The experiment was performed five times for each film type, and the results are given as average values. Water uptake was calculated using the equation:

(2) $$water\;uptake\left[\%\right]=(\frac{m_{s}-m_{i}}{m_{i}})\times 100$$

m_s_—mass of the swollen film, m_i_—initial of the completely dried film

#### 2.4.8. Antioxidant Assay (A DPPH Radical Scavenging Activity)

The 2,2-diphenyl-1-picrylhydrazyl (DPPH) radical scavenging activity of the biocomposite films was measured as described previously [8,34]. In brief, 40 mg of the PVA reference film and PVA biocomposite films regarding to proportion of dry matter of the films was weighed and placed in 10 mL flask. Water was then added, and water suspensions of the films were thus prepared. Then, 90 µL of film solutions and water (blank sample) were pipetted into 2.5 mL disposable polystyrene cuvettes, followed by the addition of 2.25 mL of a DPPH solution (40 mg L^−1^) in methanol. The cuvettes were then mixed and covered with 10 × 10 mm polyethylene lids. The reaction took place for 30 min in the dark. The antioxidant activity of the prepared solutions was examined using a Lambda UV-Vis spectrophotometer (Perkin Elmer, Waltham, MA, USA) by measuring the absorbance at a wavelength of 517 nm. Results are reported as the average of 3 measurements for each film. Results were calculated using the equation:

(3) $$RSA[\%]=\frac{A_{517nm,DPPH}-A_{517nm,S}}{A_{517nm,DPPH}}\times 100$$

A_517nm,DPPH_—absorbance at 517 nm of blank sample, A_517nm,S_—absorbance at 517 nm of sample (film) solution.

#### 2.4.9. Statistical Analysis

The results were examined for significant differences using basic statistical analysis with Statgraphics Centurion 17 software. Analysis of variance (ANOVA) and Fisher’s least significant difference (LSD) analysis with a confidence level of 95% were performed.

## 3. Results and Discussion

### 3.1. Characterization

#### 3.1.1. Thickness

**Figure 1:**
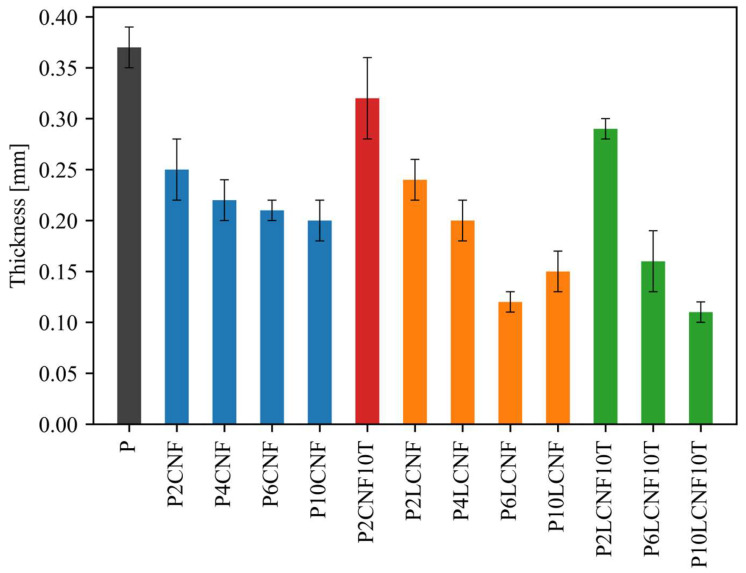
Thickness of PVA (black), PVA-CNF (blue), PVA-CNF-TA (red), PVA-LCNF (orange) and PVA-LCNF-TA (green) composite films (ANOVA, *p* < 0.0001).

Figure 1 shows the average thickness and standard deviations of the PVA reference film, the two-component PVA biocomposite films with CNFs/LCNFs and the three-component PVA biocomposite films with CNFs/LCNFs and TA. The PVA reference film (P) was the thickest. The addition of CNFs and LCNFs to the PVA matrix resulted in a lower thickness, which was probably due to the replacement of PVA with light CNFs and LCNFs. PVA biocomposite films with CNFs were slightly thicker than PVA biocomposite films with LCNFs. This was particularly observed with higher proportions of nanocellulose. This may be due to the fact that the aqueous LCNF suspension added to the PVA solution to produce the biocomposites had a lower solid content (0.7%) than the aqueous CNF suspension (1.4%). When the aqueous suspension of LCNFs was added to the PVA polymer solution, a higher water content was introduced than when PVA-CNF suspensions were used to produce biocomposite films.

#### 3.1.2. Morphology

**Figure 2:**
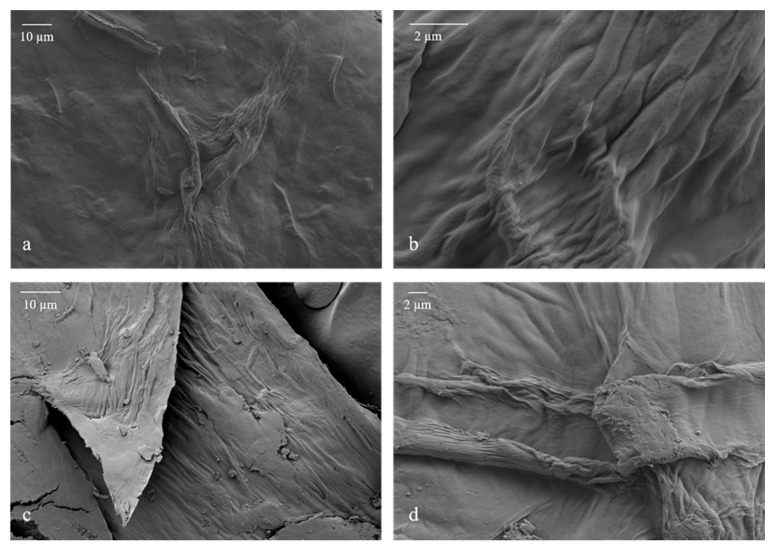
FE-SEM images of LCNF film—(**a**,**b**) and of freeze-dried LCNFs—(**c**,**d**).

Figure 2 shows FE-SEM images of LCNF film (a, b) and freeze-dried LCNF (c, d). FE-SEM was used to examine the morphology of the produced LCNFs. The presence of nanofibrils obtained with a high-pressure homogenizer was confirmed with both methods of LCNF preparation for morphology analysis, in film form and in freeze-dried form. The nanofibrils are clearly visible, with individual fibrils visible in some locations and aggregated in others.

FE-SEM was also used to investigate the dispersion of LCNFs in the PVA matrix and the rearrangement of the structure of PVA composite films after the tensile test. The morphology of the PVA reference film and the PVA-CNF biocomposite films was detailed in a previous study [8]. The PVA reference film exhibited a relatively homogeneous structure, and the FE-SEM images of the PVA-CNF biocomposite films showed the presence of individual CNFs and CNF aggregates.

**Figure 3:**
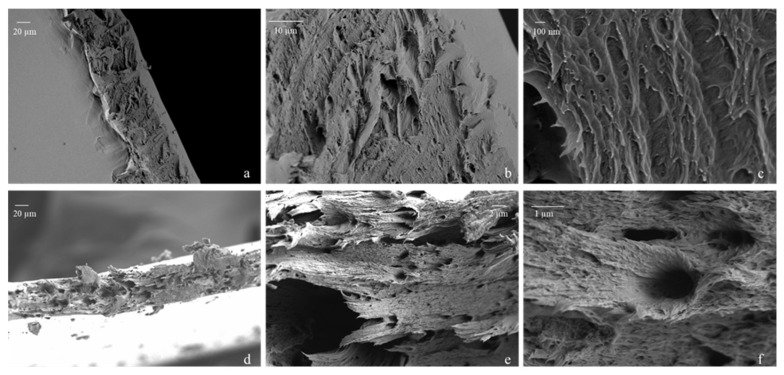
FE-SEM images of P6LCNF before the tensile test—(**a**–**c**) and after the tensile test—(**d**–**f**).

**Figure 4:**
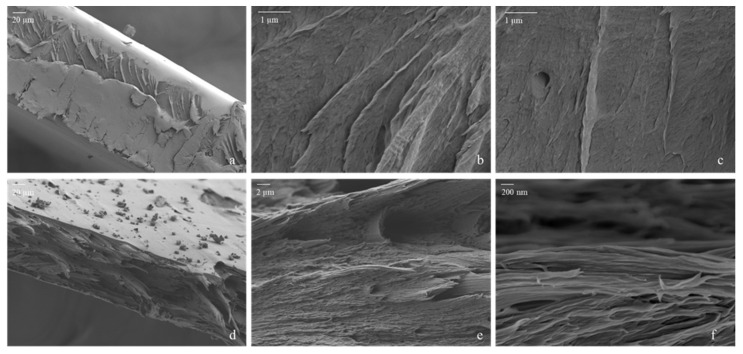
FE-SEM images of P2LCNF10T before the tensile test—(**a**–**c**) and after the tensile test—(**d**–**f**).

FE-SEM images of the surface of the P6LCNF and P2LCNF10T films were also taken (see Supplementary Figures S2 and S3), which show that the surface of the films is covered with the polymer PVA and appears to be very smooth. This can also be seen in Figure 3a (P6LCNF) and Figure 4a (P2LCNF10T), in which the smooth upper part of the film can be seen. The surfaces of the PVA-LCNF and PVA-LCNF-TA films appear to be very heavily covered with the polymer since the PVA apparently behaves like a kind of glue that coats the fibrils, which is why the surface of the films looks so smooth under an FE-SEM microscope [35]. It is likely that the nanofibrils are embedded fairly homogeneously in the PVA matrix. Images of the side profile of the film were therefore also taken in FE-SEM to gain an insight into the structure of the P6LCNF and P2LCNF10T films.

Figure 3 and Figure 4a–c show a view of the undamaged part of the P6LCNF and P2LCNF10T films, and Figure 3 and Figure 4d–f show a view of the broken part of the films (P6LCNF and P2LCNF10T) after the tensile test. As reported in a study [35] in which the addition of CNF to PVA led to an improvement in the ductility of PVA-CNF composite films, similar observations were made in our study, in which an improvement in mechanical properties was also achieved by the addition of LCNFs to the PVA. The improvement in the mechanical properties of PVA-LCNF and PVA-LCNF-TA biocomposite films can be seen from the FE-SEM images, which give us an insight into the surface of the undamaged and broken films after the tensile test. Compared with the surface of the film before the tensile test, i.e., with undamaged film, it can be seen that some LCNFs protrude from the fracture surface after the mechanical stress, and some of them are also aligned parallel to the surface of the film, which means that the addition of LCNFs in PVA leads to a material with a layered structure and, consequently, better mechanical properties compared to the PVA reference film [9,13,35].

#### 3.1.3. FTIR Analysis

FTIR spectra of LCNFs, of spruce wood from which LCNFs were formed, and of MA, which was used as a reagent for the modification and easy defibrillation of spruce wood to form LCNFs are presented in Figure S1 (Supplementary Figure S1). There are characteristic peaks for lignocellulose, which are 1423 cm^−1^ for CH_2_ scissoring motion, 1317 cm^−1^ for CH_2_ vibration, and 1024 cm^−1^ for C-O-C pyranose ring stretching [7]. The peaks at 1575 and 1510 cm^−1^ may be related to stretching vibrations in the aromatic C=C structure of residual lignin in the LCNFs [2,7]. The peak at 1718 cm^−1^ probably represents C=O stretching in the carboxyl groups of LCNFs, which was introduced into the lignocellulosic surface after pretreatment of the starting biomass with MA. The FTIR spectrum of LCNFs also shows a signal at 1208 cm^−1^, which, together with the signal at 1718 cm^−1^, may indicate the formation of an ester bond between the OH group of lignocellulose and the C=O group of MA. The FTIR spectra of spruce wood showed a characteristic peak for hemicellulose at 1733 cm^−1^; this peak was not present in the spectra of LCNFs, which may mean that the hemicelluloses were removed when the wood was treated with MA (Figure S1).

**Figure 5:**
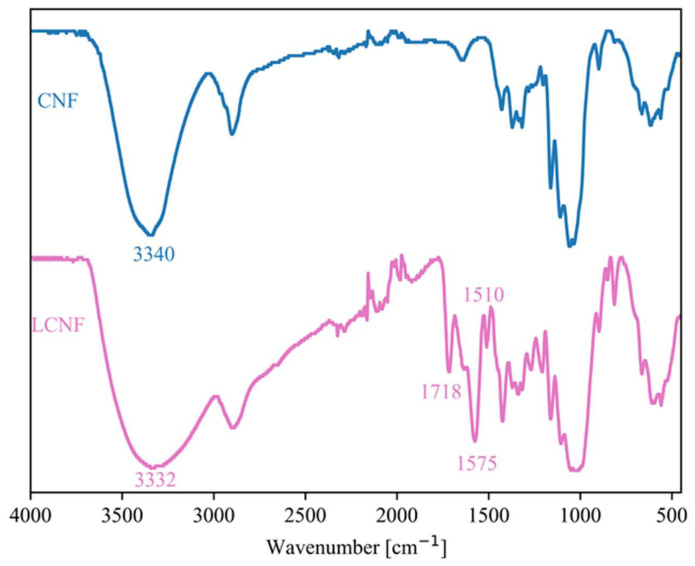
FTIR spectra of CNFs and LCNFs.

The chemical structure of the CNFs and LCNFs used to reinforce the PVA matrix was different, which was confirmed by FTIR spectroscopy. The FTIR spectra of CNFs and LCNFs are shown in Figure 5. In both spectra, a band for –OH stretching can be observed with a peak at 3340 cm^−1^ for CNFs and at 3332 cm^−1^ for LCNFs. The band for –OH stretching is broader for LCNFs than for CNFs, which is due to the presence of aromatic OH groups in the lignin structure. The spectra also differ in the FTIR range of the shorter wavenumbers. Characteristic peaks at 1575 cm^−1^ and 1510 cm^−1^ (C=C in lignin) were observed in the FTIR spectra of LCNFs, which are not present in the spectra of CNFs. The peak at 1718 cm^−1^ also appeared only in the spectra of LCNFs and may be related to the stretching of C=O in the carboxyl group on the surface of the LCNFs, which was introduced into the lignocellulose during the pretreatment of the input biomass with MA.

**Figure 6:**
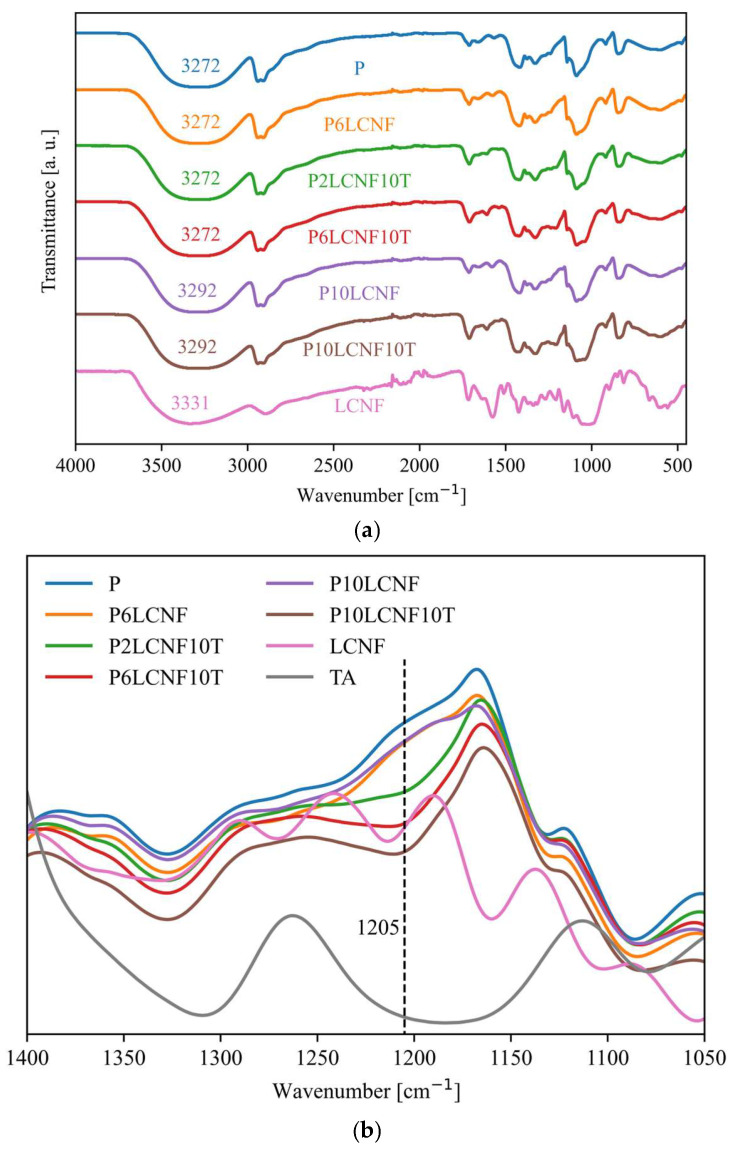
FTIR spectra of the PVA reference film (P) and PVA biocomposite films over the whole spectral range—(**a**) and in the spectral range from 1400 to 1050 cm^−1^—(**b**).

The FTIR spectra of the neat P film, P6LCNF, P10LCNF, P2LCNF10T, P6LCNF10T, and P10LCNF10T are shown in Figure 6a. The FTIR spectra of the P2LCNF and P4LCNF films are not attached and are consistent with the FTIR spectra of the P6LCNF film. Characteristic bands for PVA were observed in the spectra of all the prepared PVA composite films as -OH stretching, -CH stretching, -CH_2_ bending, C-O stretching (3272 cm^−1^, 2940 cm^−1^, 2907 cm^−1^, 1418 cm^−1^, 1088 cm^−1^) as described in a previous study [8]. In the FTIR spectra of P, P6LCNF, P2LCNF10T, P6LCNF10T, the -OH stretching was at 3272 cm^−1^, while in the spectra of films P10LCNF and P10LCNF10T this band was shifted to a higher wavenumber—3292 cm^−1^, probably indicating the formation of more intermolecular hydrogen bonds between PVA, LCNFs, and TA [2]. In a study by Zhang et al. [2], a shift of the O-H stretching vibrations to higher wavenumbers was also observed in the FTIR spectra of PVA nanocomposite films with an increasing proportion of TEMPO LCNFs in the PVA matrix. At lower concentrations of LCNFs in the PVA matrix, this shift was not observed for the oscillation of the O-H bond, probably because the concentration of LCNFs was too low. Phenols have a considerable H-bonding capacity that enables them to interact strongly with practically any hydrophilic substrate [3,8], as seen in our study with PVA and cellulose. The FTIR spectra of P2LCNF10T, P6LCNF10T, and P10LCNF10T show a peak at 1205 cm^−1^ and the FTIR spectra of LCNFs at 1208 cm^−1^ (Figure 6b). This peak was not present in other FTIR spectra (P, TA, P2LCNF; P4LCNF; P6LCNF, P10LCNF). The peak at 1205 cm^−1^, together with the peak at 1711 cm^−1^ (Figure 6a), may indicate the formation of ester bonds, which in turn may mean that the addition of TA to the PVA-LCNF system results in the formation of ester bonds between the components of the biocomposite film, in addition to hydrogen bonds.

#### 3.1.4. Tensile Test

**Figure 7:**
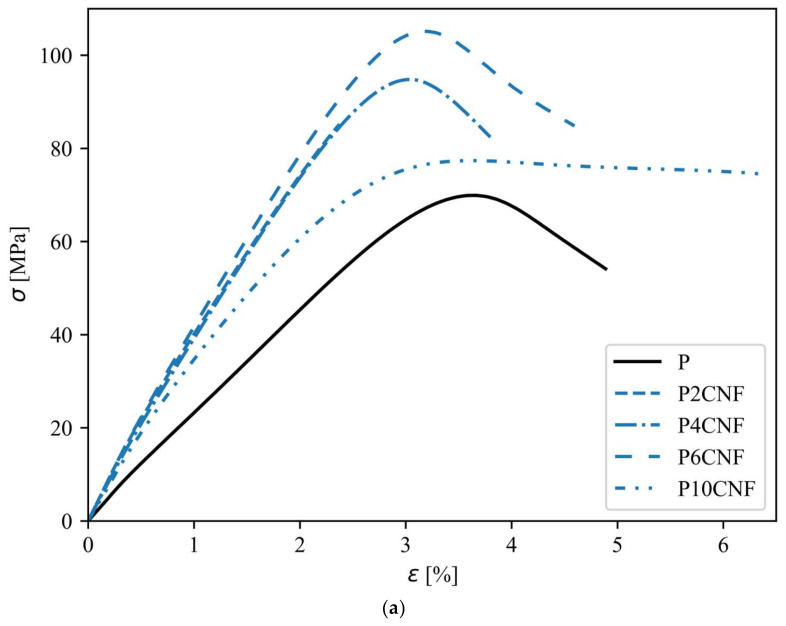

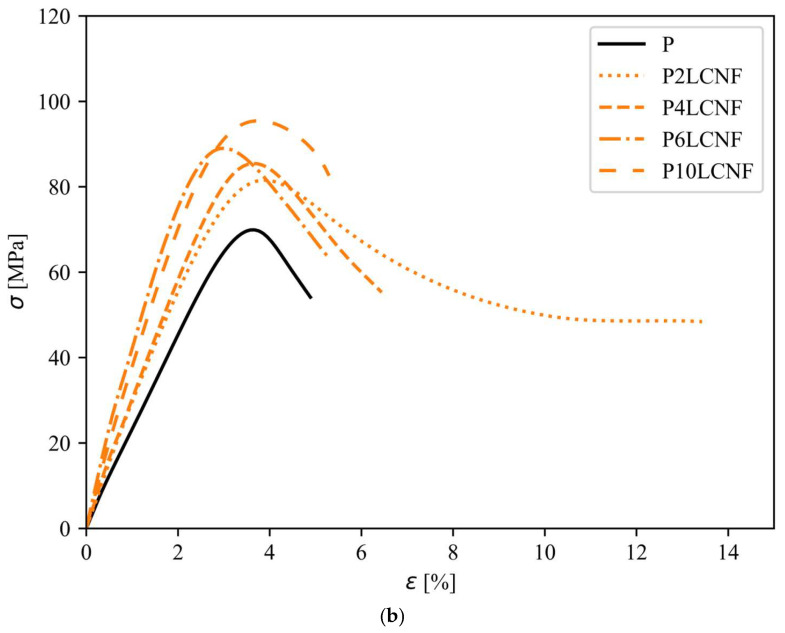
Average stress–strain curves for the PVA reference film and two-component PVA-CNF films—(**a**) and two-component PVA-LCNF films—(**b**).

The average values and standard deviations of the mechanical properties (elastic modulus E_t_, tensile strength σ_M_, elongation at break ε_tb_) for the PVA reference film and for the PVA biocomposite films with CNFs/LCNFs and with TA are listed in Table 2. Figure 7a,b show stress/strain curves representing the average curves of at least 10 specimens for the PVA reference film and the PVA-CNF/LCNF biocomposite films with 2%, 4%, 6%, and 10% nanocellulose content, with each average curve ending with the lowest elongation at break.

The addition of both types of nanofibrils, CNFs and LCNFs, to the PVA matrix resulted in PVA biocomposites with improved elastic modulus and tensile strength (Figure 7a,b), which can be attributed to the stiffness of the nanocellulose due to the extensive inter- and intramolecular hydrogen bonding with itself and the compatibility between PVA and nanofibrils (CNFs and LCNFs), as well as the homogeneous distribution of the nanofibrils in the PVA matrix [19,36]. The nanofiller must be well dispersed in the polymer matrix in order to obtain composites with better functional properties. Similar to CNFs, LCNFs also entangle and form entanglements in the structure that contribute to fiber–fiber and fiber–matrix load transfer when the material is subjected to mechanical loading, resulting in composites with better mechanical properties [19]. Strong intermolecular bonds, such as hydrogen bonds and probably ester bonds, detected by FTIR spectroscopy (Figure 6), as well as mechanical interlocking between nanofibrils, affect the stiffness and strength of the biocomposite films [13].

The results show that both types of nanofillers, CNFs and LCNFs, are suitable for reinforcing PVA. A comparison of the effects of the two nanofillers on the final mechanical properties of PVA biocomposite films was performed in this study. In terms of tensile strength and elastic modulus, the optimum addition of CNFs and LCNFs to the PVA matrix was 6%. These measured mechanical parameters were highest for P6C and P6LCNF (Table 2). The PVA biocomposites with lower CNFs and LCNFs contents also showed very high values for the elastic modulus and tensile strength. An excessive amount of nanofibrils (P10C, P10LCNF) resulted in aggregation of the nanofibrils, and such aggregates may also cause defects in the structure of the composite, making load transfer impossible and, consequently, deteriorating the mechanical properties of the biocomposite film.

At CNF concentrations up to 6%, CNFs proved to be a nanofiller with higher reinforcement potential for the PVA matrix than LCNFs since PVA-CNF films showed higher tensile strength values than PVA-LCNF films (Table 2). On the other hand, a comparison of the influence of CNFs and LCNFs on the elasticity of the formulated PVA biocomposite films showed that PVA-CNF biocomposite films were less elastic and more brittle than PVA-LCNF films. This was particularly observed at a lower LCNF loading; P2LCNF showed a 109% higher value for elongation at break than the PVA reference film and a 117% higher value for elongation at break than the P2CNF film. Despite the much higher elongation at break, the tensile strength of the P2LCNF film remained 21% higher, and the modulus of elasticity was 26% higher than the PVA reference film. In general, with the increasing addition of nanocellulose to the polymer, the tensile strength improves to a certain extent while the elongation at break decreases [13,35,37]. This trend was also observed in our study (Table 2). The elasticity of the P2LCNF film can probably be attributed to the water that remained trapped in the film during the drying of the films, and the P2LCNF film probably exhibited such a high elongation at break because water acts as a plasticizer [1]. In the case of the P2LCNF film, the water was more trapped in the film since it bonded with the PVA chains. However, with higher LCNF additions, the water could not bond as strongly with the PVA since more bonds were probably formed between the LCNFs and PVA, so the water evaporated more easily, and the films became more brittle compared to P2LCNF. A second probable reason for the high elasticity of the P2LCNF film was that the LCNF concentration in the P2LCNF formulation was just right so that the nanofibrils were uniformly and homogeneously distributed in the PVA matrix. There were, therefore, neither too many of them nor many LCNF agglomerates, as can occur in formulations with a higher LCNF content in the PVA matrix, in which the fibrils are connected by many more intermolecular H-bonds, giving the final film strength and fragility at the same time.

**Figure 8:**
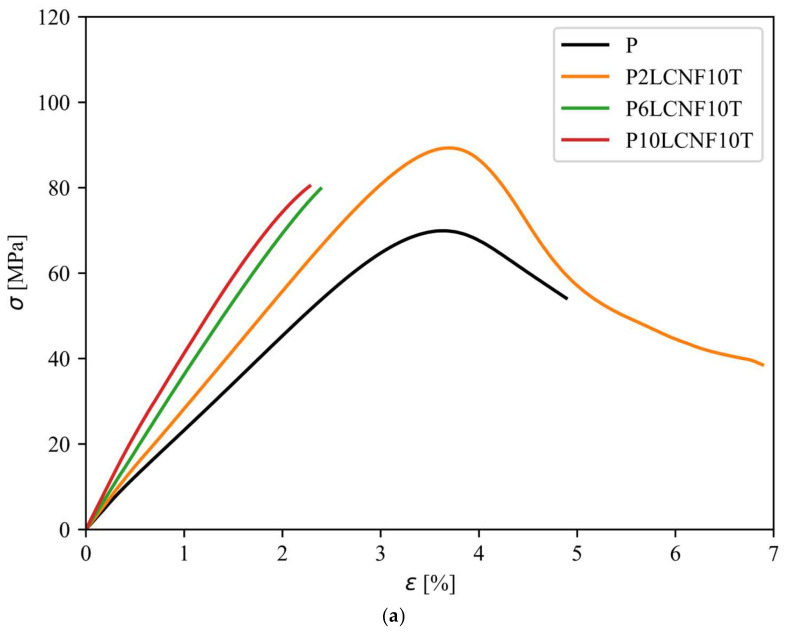

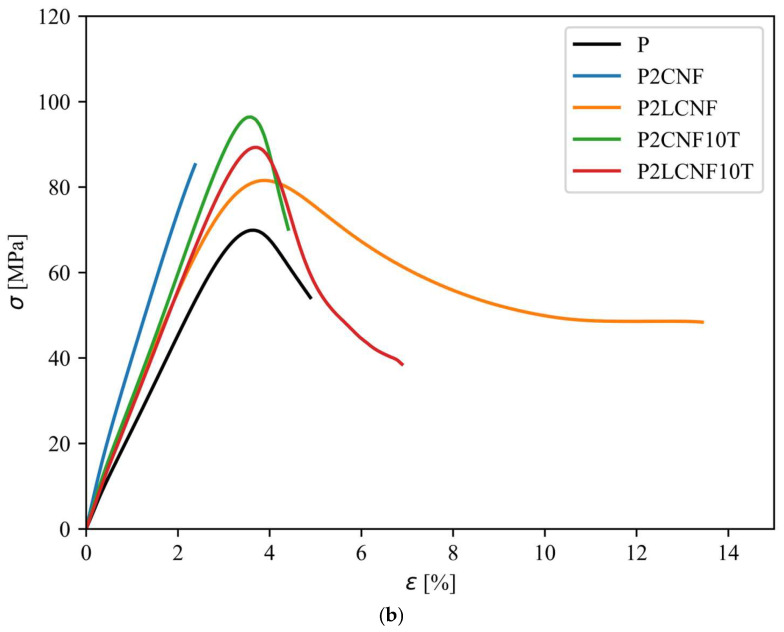
Average stress–strain curves for the PVA reference film and three-component PVA-LCNF-TA films—(**a**) and for the PVA reference film and PVA biocomposite films with CNF or LCNF and TA—(**b**).

The curves in Figure 8a,b are the average stress–strain curves of the PVA reference film and PVA biocomposite films with CNFs/LCNFs and TA, each ending at the lowest elongation at break. As shown in a previous study [8], the addition of higher TA concentrations resulted in more cross-linked PVA biocomposite films and, furthermore, in more brittle films, which was also evident here as the values of elongation at break decreased after the addition of TA to PVA-CNF and PVA-LCNF system (Table 2). The cross-linking of nanocellulose, TA, and PVA was also evident in the FTIR analysis, in which hydrogen and ester bonds were confirmed.

As reported in a previous study [8], the addition of TA resulted in more brittle films. The P2LCNF, P6LCNF, and P10LCNF films exhibited a more than twice higher value for elongation at break than the three-component biocomposite films (P2LCNF10T, P6LCNF10T, and P10LCNF10T) for the same amount of LCNFs in the PVA matrix. Comparing the tensile strength of P2LCNF and P2LCNF10T showed that TA also contributed to a higher tensile strength of the final composite film, while this was not observed in the case of CNFs as nanofillers (Table 2).

Tensile strain is one of the most important properties in relation to the use of these films as packaging materials, so it is crucial to obtain composite films with all three tensile properties improved—tensile strength, modulus of elasticity, and elongation at break [2]. Among the two-component PVA films with CNF/LCNF, the P6LCNF film exhibited a higher value of elastic modulus, a very high value of tensile strength, and the value of elongation at break also remained quite similar to that of the PVA reference film. Compared with the PVA reference film, the elastic modulus was improved by 92% and the tensile strength by 39% (Table 2). The addition of higher LCNF contents to the PVA matrix is likely to result in the formation of larger LCNF aggregates, which means that the LCNFs are not as well dispersed in the polymer matrix since they are at lower concentrations, leading to a reduction in the mechanical properties of the final biocomposite film [2].

Among the three-component films with LCNFs (Figure 8a), the P2LCNF10T film was the most suitable for use in packaging applications in terms of mechanical properties since the addition of 2% LCNFs and 10% TA maintained the flexibility and elasticity of the film, while the modulus of elasticity and tensile strength were greatly improved (the elongation at break remained the same as that of the PVA reference film). LCNFs tend to agglomerate at higher loadings, which affects the tensile strength of the P10LCNF10T film, which was lower than the tensile strength of the other two three-component films. Agglomeration of LCNFs may lead to a breakdown in the interactions among the components of the composite, resulting in weaker areas in the film [21].

The addition of 10% TA to the PVA matrix with 2% LCNFs improved the tensile strength of the final composite by 32% compared to the PVA reference film, while the elasticity of the biocomposite, i.e., the elongation at break value, remained almost the same as for the PVA reference film. However, the addition of 10% TA to the PVA matrix with 2% CNFs improved the tensile strength by 43%, and the elongation at break decreased by 15% compared to the PVA reference film (Table 2). As in the case of two-component PVA biocomposite films, also among the three-component composite films (with the addition of nanocellulose and TA), biocomposites using LCNFs as a nanofiller proved to be more elastic and less brittle than biocomposites with CNFs, which can be attributed to the more amorphous structure of LCNFs compared to CNFs (Figure 8b).

#### 3.1.5. Thermal Analysis

The thermal degradation of lignocellulosic fibers is a complex process involving a series of competing/sequential reactions. The thermal stability depends mainly on the chemical composition, fiber size, crystal structure, and the number of inter- and intramolecular hydrogen bonds [17].

In polymer composites, the dispersion of the filler in the polymer matrix and the interfacial adhesion between the matrix and the filler are other important factors that influence thermal stability [38].

The addition of fillers—nanocellulose and TA—affected the thermal properties of PVA biocomposite films (Table 3). LCNFs had a stronger effect on the T_g_ than CNFs since, in the case of the P10LCNF biocomposite, the T_g_ was 6 °C higher than with the PVA reference film, and in the case of the P10CNF biocomposite, i.e., with the same amount of nanocellulose, the T_g_ was 3 °C higher than with the PVA reference film. When TA was added to the PVA matrix, there were more significant differences in the change in T_g_ of the biocomposites. With a 2% proportion of nanocellulose (CNFs and LCNFs) and 10% TA, in the biocomposite P2CNF10T and in the biocomposite P2LCNF10T, the T_g_ was higher than without the addition of TA with the same proportion of nanocellulose. The T_g_ was 83 °C for both biocomposites and thus 8 °C higher than for the PVA reference film. The results obtained are consistent with a previous study [8], in which the addition of TA to the PVA matrix had a greater effect on the T_g_ of the biocomposites than the addition of nanocellulose. With the increasing addition of LCNFs to the PVA matrix at 10% TA addition, the T_g_ for the biocomposites produced increased and amounted to 88 °C in the case of the P10LCNF10T film. The higher T_g_ values can be attributed to intermolecular interactions between the building blocks of the PVA biocomposite films, which restrict the movement of the PVA chains and reduce their flexibility [39]. This behavior is also consistent with the results of an earlier study in which we investigated the thermal properties of PVA-CNF-TA biocomposite films [8].

With an increasing addition of nanocellulose (CNFs, LCNFs), the T_m_ for PVA biocomposites decreased (Table 3). This was more pronounced for LCNFs than for CNFs and more pronounced for three-component PVA biocomposites than for two-component PVA biocomposites. The decrease in T_m_ coincides with a decrease in crystallinity, which in turn was more pronounced for the three-component PVA biocomposites. Interactions between the components of the PVA biocomposite (hydrogen bonds) can hinder the arrangement of the PVA molecules and thus also reduce the crystallinity [8,40].

Up to 600 °C, PVA biocomposite films decomposed in three stages, as already described in the article by Osolnik et al. [8]. The first weight loss occurred in the temperature range from room temperature to 200 °C and was mainly due to the evaporation of water; the second weight loss was due to the decomposition of the main molecular chains in the PVA.

PVA-LCNF biocomposites started to decompose thermally at lower T values than PVA-CNF biocomposites, at about 12 °C lower T values with the same nanocellulose content (Table 4). This can be attributed to the fact that the structure of LCNFs is more amorphous than the structure of CNFs due to the presence of amorphous lignin. Ordered, crystalline regions can act as barriers and hinder heat diffusion in the structure, while heat diffusion is easier in disordered, amorphous regions [41]. Furthermore, in addition to lignin and cellulose, LCNFs may also contain a certain amount of hemicellulose, which is considered to be less thermally stable than lignin and cellulose [38], which naturally affects the poor thermal stability of the PVA-LCNF biocomposite compared to PVA-CNF biocomposites.

The addition of CNFs to the PVA matrix had little effect on the T_onset_ and T_max_ of the PVA-CNF biocomposites compared to the PVA reference film, with the exception of the P10CNF biocomposite film, with which the T_onset_ decreased by 8 °C compared to the PVA reference film, while the T_max_ was only 2 °C lower compared to the PVA reference film. The addition of LCNFs to the PVA matrix led to a more significant reduction in the T_onset_ and T_max_ of the PVA-LCNF biocomposite films, with the greatest reduction in the T_onset_ and T_max_ of the P10LCNF biocomposite film also occurring with a 10% addition of nanocellulose. It should be emphasized here that lignin decomposes more slowly and over a wider temperature range [17,42].

The addition of TA to the PVA-LCNF system resulted in a slightly higher T_onset_ and a slightly lower T_max_ for all PVA-LCNF-TA biocomposite films compared to the PVA reference film. The higher T_onset_ value in the case of PVA-LCNF-TA biocomposites is probably due to the compatibility and interactions among the components of the biocomposite [39].

The T_max_ decreased with increasing LCNF content in PVA-LCNF-TA systems. The P2CNF10T biocomposite film showed the same T_onset_ value as the PVA reference film and a slightly higher T_max_ than the PVA reference film.

#### 3.1.6. Contact Angle

**Figure 9:**
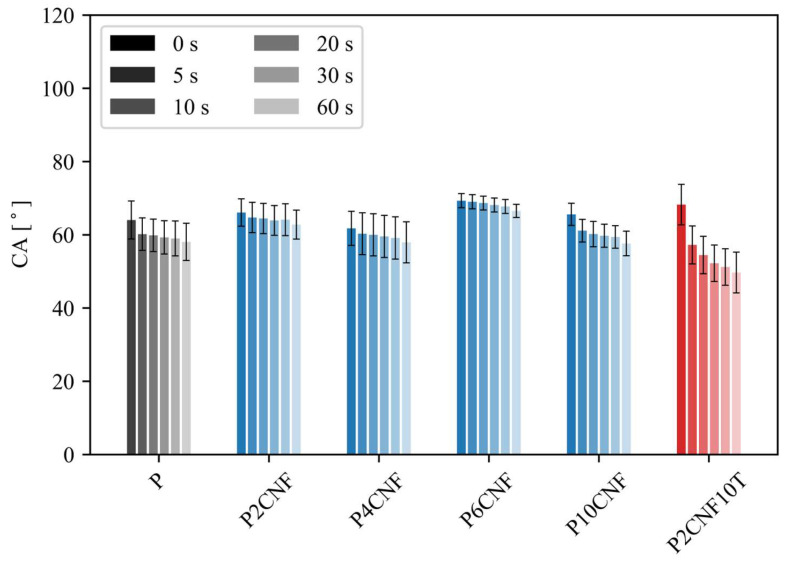
Contact angle over time (0–60 s) for the PVA reference film (grey) and PVA biocomposite films with CNFs (blue) and with both CNFs and TA (red) (ANOVA, *p* < 0.0001).

**Figure 10:**
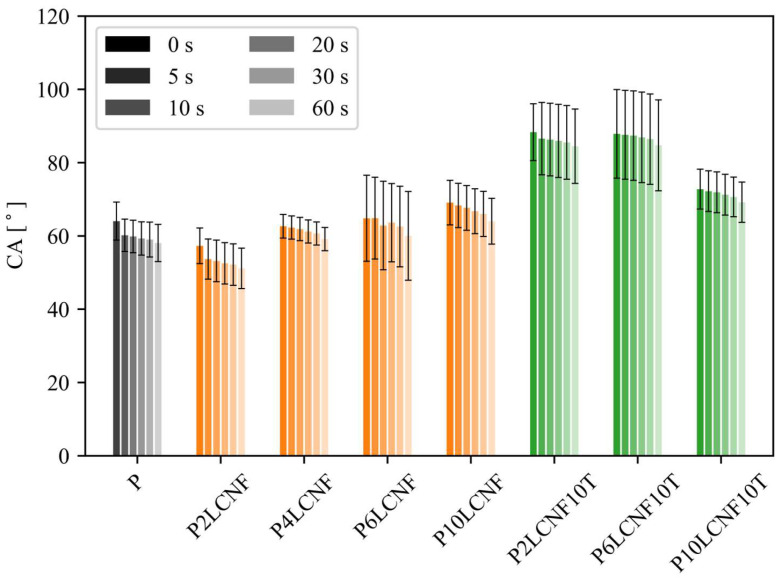
Contact angle over time (0–60 s) for the PVA reference film (grey) and PVA biocomposite films with LCNFs (orange) and with both LCNFs and TA (green) (ANOVA, *p* < 0.0001).

Figure 9 and Figure 10 show the contact angle as a function of time from 0 s to 60 s for the PVA reference film and all PVA two-component films with CNFs and three-component films with CNFs and TA. The addition of CNFs had no significant effect on reducing the surface hydrophilicity of the PVA biocomposite films with CNFs, while the P6CNF films had an approximately 5° higher contact angle value throughout the 60 s measurement compared to the PVA reference film. The P2CNF10T film also had a higher contact angle of about 5° at the beginning, but the contact angle value at the end was lower than that of the PVA reference film.

The addition of LCNFs alone to the PVA matrix also had virtually no effect on increasing the contact angle of the water droplet on the surface of the PVA-LCNF composites; exceptionally, slightly higher contact angles were observed for the PVA film with 10% LCNFs (Figure 10). Slightly lower contact angles were determined for the P2LCNF film, which was probably due to water remaining in the film. LCNFs were added as a 0.7% suspension of LCNFs in water to all suspensions produced for the cast films. The water in the P2LCNF film was probably more strongly retained and did not evaporate, as well as in other formulations with a higher LCNF content. This was also evident in the mechanical results: the P2LCNF film was the most elastic among all films produced. Although more water was added to the suspensions with a higher LCNF content, this water evaporated more easily when the films dried, probably because the nanofibrils in the biocomposite formulations with higher LCNF concentrations were more strongly bonded to each other and to the polymer matrix and the water was not bound as strongly in the film.

In contrast, larger differences in contact angles were observed for the three-component biocomposite systems PVA-LCNF-TA compared to the PVA reference film; especially for P2LCNF10T and P6LCNF10T, the contact angle values were more than 20° higher than for the PVA reference film. The above films exhibited an almost hydrophobic character, lacking only about 2° to 90°, which is the criterion for hydrophobicity [43]. The higher values of the contact angle of the water droplet on the surface of the PVA biocomposite films were probably due to the interactions among all the components of the film. As revealed by FT-IR spectroscopy, OH groups on PVA form hydrogen bonds between polar functional groups (OH, COOH) on LCNFs and TA, which means that there are fewer OH groups on the PVA chain in such a connected system, which is the reason for the decrease in hydrophilicity, especially in P2LCNF10T and P6LCNF10T films compared to the PVA reference film. The contact angle values of the water droplet on the surface of the P10LCNF10T film were lower than that of the other three-component biocomposites, probably due to an excessive amount of fillers, especially LCNFs, as shown by the mechanical tests. LCNFs tend to agglomerate at higher concentrations and, moreover, probably do not form bonds with the free OH groups of PVA, so the hydrophilicity of the composite could not be reduced.

#### 3.1.7. Water Uptake

**Figure 11:**
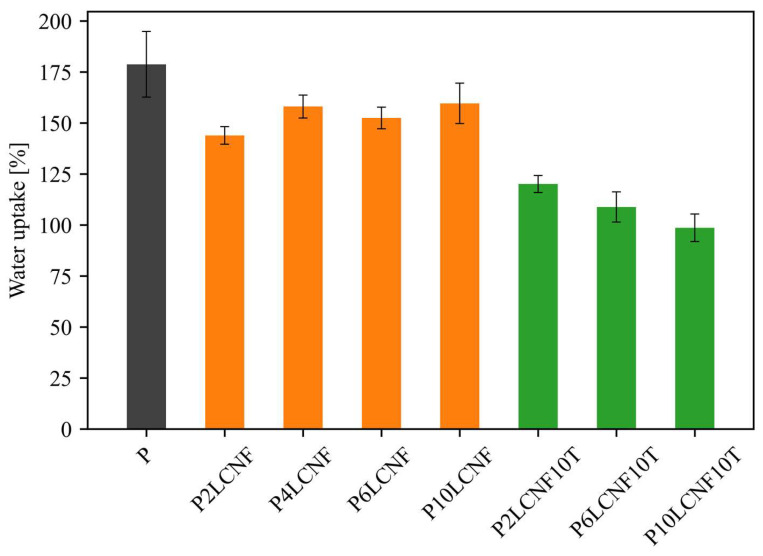
Water uptake for the PVA reference film (grey) and PVA biocomposite films with LCNFs (orange) and with both LCNFs and TA (green) after 1 h of soaking films in water (ANOVA, *p* < 0.0001).

Figure 11 shows the water uptake for the PVA reference film and the PVA biocomposite films with LCNFs and TA after the films were soaked in water for one hour. The addition of LCNFs to the PVA matrix slightly improved the water uptake of the biocomposite film compared to the PVA reference film, similar to PVA-CNF films, which was confirmed by the results of a previous study [8]. P10LCNF showed the highest water uptake among the PVA-LCNF systems after one hour of soaking. When the distribution of LCNFs in the PVA matrix is homogeneous, and the LCNF content is not too high, there are probably no larger LCNF aggregates, which probably means that the hydrophobic lignin in the LCNFs can thus cause a lower water uptake in PVA-LCNF composite systems compared to a base polymer matrix. As in the determination of the hydrophilicity or hydrophobicity of the surface of PVA biocomposite films, the addition of TA to the PVA-LCNF system proved to be very successful, which was also evident here since the PVA-LCNF-TA biocomposite films absorbed more than 50% less water than the PVA reference film. The P10LCNF10T film absorbed more than 70% less water than the PVA reference film. The interactions among all three components in the P10LCNF10T film, confirmed by FTIR spectroscopy, probably formed a 3D cross-linked structure that made it difficult for water to reach the structure of the three-component film.

#### 3.1.8. Antioxidant Assay (A DPPH Radical Scavenging Activity)

In materials used for packaging purposes, an antioxidant activity of the material is also desired to extend the shelf life of the packaged product by protecting the product from oxidation processes [44]. The antioxidant activity of the prepared films was therefore investigated by the DPPH method. As shown in Table 5, the PVA reference film and the two-component PVA films with CNFs and LCNFs were not antioxidant active, while only P10LCNF showed some but negligible antioxidant activity. As described in a previous study [8], tannic acid is antioxidant active in both free and bound forms. All three-component films produced showed a very similar antioxidant activity of around 86%, which means that they could be a potential candidate in the field of active packaging materials. With increasing LCNF content, the antioxidant activity of the three-component films did not increase.

## 4. Conclusions

Like CNFs, LCNFs are a suitable natural alternative to petroleum-based fillers for reinforcing biodegradable polymers such as PVA. In the study, LCNFs were successfully obtained by esterification of spruce wood with maleic anhydride and subsequent mechanical defibrillation. LCNFs and CNFs were successfully incorporated into the PVA polymer matrix to obtain two-component PVA-CNFs and PVA-LCNFs biocomposite films, as shown by the results of FTIR analysis and tensile test. The PVA-CNFs and PVA-LCNFs biocomposite films showed significantly improved mechanical properties compared to the PVA reference film. The optimum percentage of both types of nanocellulose to achieve the strongest biocomposite films was 6%. At lower weight percentages, the addition of LCNFs contributed significantly to the higher elasticity of the biocomposite. Three-component biocomposite films were prepared by adding the biologically active TA to the PVA/CNFs and PVA/LCNFs system. TA was not only antioxidant active but also acted as a cross-linking agent and influenced the mechanical properties of the biocomposites produced, as the tensile strength of the three-component biocomposites was largely maintained or even improved. TA also had an effect on the thermal properties of the biocomposites. The three-component biocomposites exhibited a higher glass transition temperature compared to the PVA reference film. The addition of TA to the PVA-LCNF system also resulted in a slightly higher Tonset for the PVA-LCNF-TA biocomposite films at all LCNF proportions. The compatibility of all three building blocks, PVA, LCNFs, and TA, with the appropriate proportion of fillers, was also reflected in the water resistance results of the three-component biocomposite films produced.

The cross-linked 3D structure of three-component PVA-LCNF-TA biocomposites is the key to producing biocomposite films with improved functional properties. Both CNFs and LCNFs are suitable nanofillers for the PVA matrix which, in combination with the addition of biologically active TA, can enable the production of packaging films with antioxidant properties for use in various packaging applications.

## Figures and Tables

**Table 1:** Film labels and composition of films.

Film Type	Film Label	% CNFs	% LCNFs *	%TA *
PVA	P	0	0	0
PVA + 2% CNFs	P2CNF	2	0	0
PVA + 4% CNFs	P4CNF	4	0	0
PVA + 6% CNFs	P6CNF	6	0	0
PVA + 10% CNFs	P10CNF	10	0	0
PVA + 2% CNFs + 10% TA	P2CNF10T	2	0	10
PVA + 2% LCNFs	P2LCNF	0	2	0
PVA + 4% LCNFs	P4LCNF	0	4	0
PVA + 6% LCNFs	P6LCNF	0	6	0
PVA + 10% LCNFs	P10LCNF	0	10	0
PVA + 2% LCNFs +10% TA	P2LCNF10T	0	2	10
PVA + 6% LCNFs + 10% TA	P6LCNF10T	0	6	10
PVA + 10% LCNFs + 10% TA	P10LCNF10T	0	10	10

* % of CNF, LCNF, and TA on the dry weight of PVA.

**Table 2:** Tensile properties of the PVA reference film (P) and PVA biocomposite films (ANOVA, *p* < 0.0001).

Film Type	E_t_ [MPa]	σ_M_ [MPa]	ε_tb_ [%]
P	2552 ± 233	68.6 ± 10.2	8.5 ± 2.5
P2CNF	4612 ± 555	97.6 ± 9.4	8.8 ± 1.0
P4CNF	4348 ± 451	96.4 ± 5.6	7.9 ± 1.6
P6CNF	4676 ± 430	105.1 ± 8.7	6.1 ± 0.9
P10CNF	4003 ± 510	78.1 ± 4.7	10.9 ± 2.3
P2CNF10T	3473 ± 283	98.1 ± 4.0	7.2 ± 0.8
P2LCNF	3227 ± 534	83.1 ± 5.3	17.8 ± 2.0
P4LCNF	3427 ± 318	87.1 ± 8.2	9.1 ± 1.2
P6LCNF	4898 ± 702	95.6 ± 12.3	8.0 ± 1.0
P10LCNF	4040 ± 502	91.4 ± 7.9	6.4 ± 0.6
P2LCNF10T	3086 ± 299	90.7 ± 5.0	8.6 ± 1.0
P6LCNF10T	3725 ± 842	93.8 ± 11.4	3.5 ± 0.9
P10LCNF10T	4539 ± 512	86.9 ± 12.4	3.3 ± 0.6

**Table 3:** Summary of glass transition temperature (Tg), melting temperature (Tm), and crystallinity (X_c_^DSC^) of the PVA reference film and PVA biocomposite films with CNFs, LCNFs, and TA.

Film Type	Tg [°C]	Tm [°C]	X_c_^DSC^ [%] ^a^
P	75	221	42
P2CNF	78	221	40
P4CNF	75	220	38
P6CNF	76	222	39
P10CNF	78	219	38
P2CNF10T	83	208	31
P2LCNF	76	219	43
P4LCNF	78	219	41
P6LCNF	80	218	40
P10LCNF	81	216	36
P2LCNF10T	83	215	31
P6LCNF10T	87	210	26
P10LCNF10T	88	207	30

^a^ Calculated by Equation (1).

**Table 4:** T_onset_ and T_max_ for the PVA reference film and PVA biocomposite films with CNFs, LCNFs, and TA.

Film Type	T_onset_ [°C]	T_max_ [°C]
P	298	364
P2CNF	299	366
P4CNF	297	366
P6CNF	294	366
P10CNF	290	362
P2CNF10T	298	366
P2LCNF	285	356
P4LCNF	284	335
P6LCNF	282	332
P10LCNF	278	327
P2LCNF10T	303	361
P6LCNF10T	303	350
P10LCNF10T	302	340

**Table 5:** Radical scavenging assay of PVA and PVA biocomposite films.

Film Type	RSA [%]	s_d_ [%]
P	0.0	0.0
P2CNF	0.0	0.0
P4CNF	0.0	0.0
P6CNF	0.0	0.0
P10CNF	0.0	0.0
P2CNF10T	85.4	1.2
P2LCNF	0.0	0.0
P4LCNF	0.0	0.0
P6LCNF	0.0	0.0
P10LCNF	1.8	1.0
P2LCNF10T	86.7	2.4
P6LCNF10T	85.4	1.0
P10LCNF10T	86.8	0.6

## Data Availability

The original contributions presented in this study are included in the article/supplementary material. Further inquiries can be directed to the corresponding author.

## Supplementary Materials

The following supporting information can be downloaded at: https://www.mdpi.com/article/10.3390/polym17010016/s1, Figure S1: FTIR spectra of spruce wood, MA and LCNFs; Figure S2. FE-SEM images of P6LCNF surface; Figure S3. FE-SEM images of P2LCNF10T surface.
