# Supplementary material for: (Ligno)Cellulose Nanofibrils and Tannic Acid as Green Fillers for the Production of Poly(vinyl alcohol) Biocomposite Films
Source: Polymers (Basel). 2024 Dec 25;17(1):16. doi: 10.3390/polym17010016 (PMC11723416; doi:10.3390/polym17010016)
Supplement: Supplementary file 1 [file polymers-17-00016-s001.zip › polymers-3369750-supplementary.pdf]

Supplement - Figures

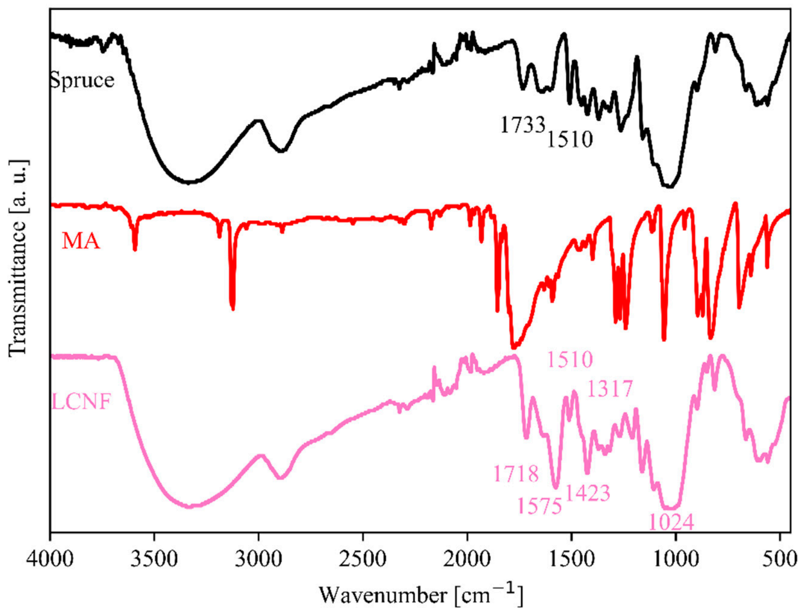

Figure S1. FTIR spectra of spruce wood, MA and LCNFs.

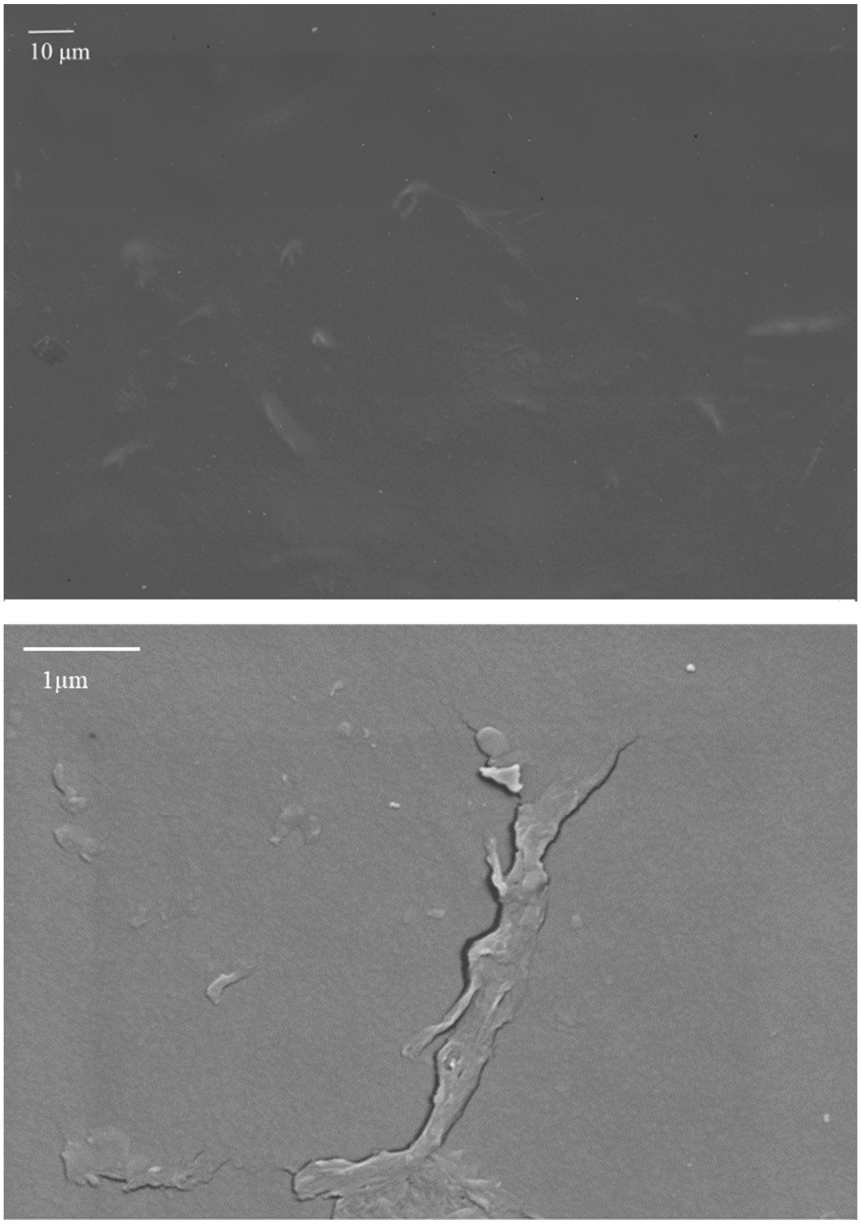

Figure S2. FE-SEM images of P6LCNF surface.

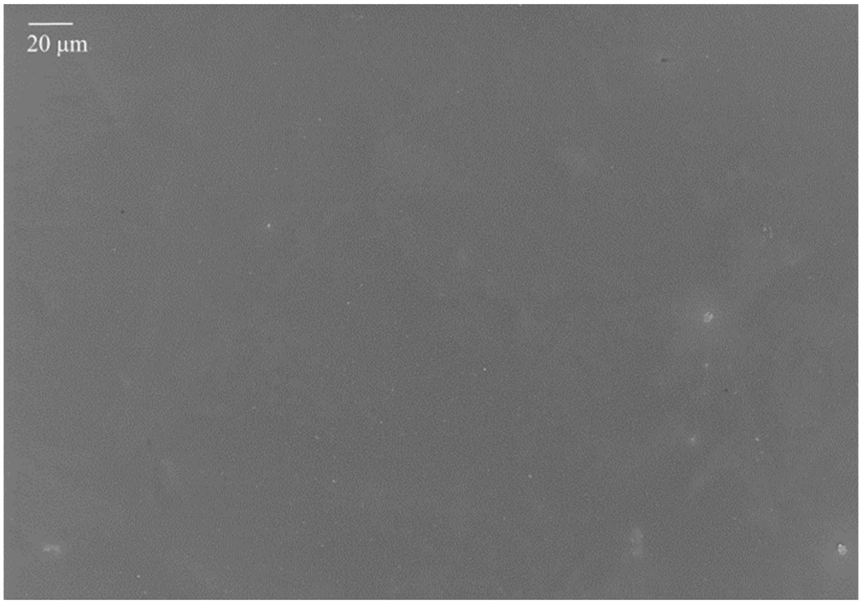

Figure S3. FE-SEM images of P2LCNF10T surface.
